# Photochemical Methods to Study the Radical-Induced Degradation of Anion-Exchange Membranes

**DOI:** 10.3390/membranes15100305

**Published:** 2025-10-07

**Authors:** Panna Solyom, Thomas Nauser, Tamas Nemeth

**Affiliations:** 1 Department of Sustainable Energy Technology, SINTEF Industry, 7034 Trondheim, Norway; panna.solyom@sintef.no; 2 Laboratory of Inorganic Chemistry, ETH Zurich, Vladimir-Prelog-Weg 1, 8093 Zurich, Switzerland; nauser@inorg.chem.ethz.ch

**Keywords:** fuel cells, electrolysers, radical reactions, anion exchange membranes, photochemical reactions

## Abstract

We adapted two photochemical methods to generate radicals and assess their impact on anion exchange membrane stability, independent of base-induced degradation. Through the exposure of aqueous solutions of potassium nitrite or suspensions of TiO_2_ to UV light at 365 nm, we generated hydroxyl radicals or a combination of hydroxyl and superoxide radicals. The methods’ applicability to anion exchange membranes (AEMs) is demonstrated on three commercial AEMs: PiperION-40, FM-FAA-3-PK-75, and PNB-R45. Changes in ion-exchange capacity, along with FT-IR and NMR analyses, revealed significant degradation in thinner, non-reinforced membranes, while thicker and reinforced membranes showed greater resistance. We attribute this to the limited penetration depth of highly reactive radicals into the membrane. Both methods are practical and inexpensive tools for benchmarking AEM stability against radical attack.

## 1. Introduction

Energy security and global warming have become a fundamental challenge of humanity. Global efforts are increasingly focused on replacing traditional fossil fuels with renewable energy sources and advancing electrochemical technologies for efficient energy storage and conversion. Water electrolysers (ELs) and fuel cells (FCs) are in the centre of significant attention as promising devices to balance the seasonality of renewable energy production. FCs, which efficiently convert the chemical energy of high-density fuels like hydrogen directly into electricity with zero CO_2_ emissions, offer a promising solution to decarbonise the transport sector, a major contributor to global warming and responsible for nearly one-third of global greenhouse gas emissions [1].

The high capital expenditure (CAPEX) associated with state-of-the-art proton exchange membrane (PEM) FCs and ELs stands as an obstacle to widespread commercialisation. It is caused by the use of platinum group metal (PGM) catalysts and perfluorinated membranes [2]. As a result, there is growing interest in complementary anion exchange membrane (AEM) technology, which operates under alkaline conditions and offers several advantages over PEMs: (1) kinetically more favourable oxygen evolution (OER) and reduction reactions (ORRs) that reduce activation losses, (2) the possibility to use inexpensive non-precious metal catalysts, (3) sustainable membrane alternatives to traditional perfluorinated polymers, and (4) the use of inexpensive cell components due to the less corrosive operating environment [3].

However, the widespread application of both AEMFCs and AEMWEs is currently hindered by the rapid degradation of AEMs under alkaline conditions; this challenge is further exacerbated at low hydration levels [4]. In PEMs, chemical degradation is mainly caused by the electrophilic attack of oxidising radicals formed during FC operation [5]. In AEMs, however, it is predominantly caused by the nucleophilic attack of OH^−^ [6]. Several OH^−^-induced degradation pathways for AEMs have been proposed, including (1) Hofmann elimination, (2) nucleophilic substitution, and (3) the formation of ylides, which may undergo further rearrangement via Stevens or Sommelet–Hauser mechanisms [6]. These detrimental processes can occur simultaneously, significantly compromising membrane stability and prompting extensive research efforts aimed at enhancing the alkaline durability of AEMs.

Several studies have reported accelerated degradation under oxygen-saturated conditions, suggesting that reactive oxidising species (ROS) play a significant role in the degradation mechanisms of AEMs. Advancements in polymer backbones, quaternary ammonium headgroups, and crosslinking strategies have brought about a significant improvement in the alkaline stability of AEMs; however, strategies to improve radical-induced degradation remain limited [6]. Parrondo et al. were the first to observe a significantly higher loss in ion-exchange capacity during ex situ stability tests of poly(*p*-phenylene oxide)-based AEMs performed under oxygen-saturated conditions compared to nitrogen degassed ones, highlighting the detrimental role of oxidative stress in AEM degradation [7]. Experiments involving spin trapping and the use of a fluorescent dye are interpreted by the time-dependent formation of various ROS, such as HO^•^, HOO^•^, and O_2_^•−^ [7,8].

The ex situ stability test conducted by Espiritu et al. also indicates increased IEC loss in vinylbenzyl chloride-grafted low-density polyethylene-based AEMs when exposed to oxygen [9].

Wierzbicki and colleagues were the first to detect in situ radical formation in various AEMs by operating a micro-AEM-FC within an Electron Paramagnetic Resonance (EPR) setup. This enabled the spin trapping of HO^•^ and HOO^•^ radicals on the cathode side and H^•^ radicals on the anode side [10]. Subsequent experiments revealed that radical formation occurs for both PGM-based and PGM-free electrocatalysts [11]. Under the premise that radicals are involved in the degradation of AEMs, it becomes essential to evaluate the ex situ stability of novel AEMs before subjecting them to time- and resource-intensive in-device testing.

To assess the alkaline stability of AEM headgroups or fabricated AEMs, a widely adopted approach involves exposing the materials to concentrated alkaline solutions, typically 1–12 M potassium hydroxide (KOH) or sodium hydroxide (NaOH), at elevated temperatures, i.e., 80–160 °C [12].

In contrast, in ex situ degradation tests of PEMs, where degradation is driven by radicals, Fenton’s reaction is the most common method to form oxidising radicals, HO^•^ and HOO^•^ via Reactions (1)–(3), and evaluate stability.

Fe^2+^ + H_2_O_2_ → Fe^3+^ + HO^•^ + OH^−^ *E*°(HO^•^, H^+^/H_2_O) = 2.73 V (1)

HO^•^+ H_2_O_2_ → H_2_O + HOO^•^ (2)

Fe^3+^ + H_2_O_2_ → Fe^2+^ + HOO^•^ + H^+^ *E°*(HOO^•^, H^+^/H_2_O_2_) *=* 1.46 V (3)

While it may seem straightforward to subject AEMs to Fenton’s test to benchmark oxidative stability [13], at pH values above 5, highly oxidising Ferryl radicals form rather than HO^•^ and HOO^•^ [14,15]. Reactivity and selectivity differ between Ferryl and HO^•^. Fenton chemistry, therefore, promotes misleading results in the study of the HO^•^ radical-mediated degradation of AEMs.

Alternative approaches to generate radicals include the thermal decomposition of dilute aqueous H_2_O_2_ solutions at elevated temperatures [16], or using UV activation. There are several limitations with these methods [17]. First, the thermal decomposition of H_2_O_2_ is not an efficient way to generate radicals [18]. While radical formation is possible via Reaction (4), at elevated temperatures, the process is dominated by non-radical multistep thermal decomposition pathways (Reaction (5)). Second, while the UV activation of H_2_O_2_ can effectively generate radicals, it requires irradiation at wavelengths below 300 nm. At these wavelengths, the direct photochemistry of aromatic AEMs will certainly be non-negligible and may even constitute the main reaction pathway. Moreover, the stability of H_2_O_2_ is significantly lower under alkaline conditions compared to acidic conditions, yielding oxygen as a stable decomposition product instead of the radicals HO^•^ and HOO^•^.

H_2_O_2_ → 2 HO^•^ radical path(4)

H_2_O_2_ →→ HO^•^ non-radical path(5)

A recently developed protocol enables the simultaneous assessment of the base- and radical-induced degradation of AEMs by immersion in oxygen-saturated aqueous alkaline solutions at elevated temperatures [19]. While this method offers a more realistic simulation of operational conditions, it presents several limitations. First, the rate of radical formation is highly dependent on the concentration of dissolved oxygen. It decreases with both increasing temperature and hydroxide concentration due to the salting-out effect [20]. Second, because the protocol uses concentrated hydroxide solutions, it is not possible to distinguish between base-induced degradation and radical-induced degradation.

The concept of water radiolysis as a technique for the selective production of specific radical species, using pulse- [21], gamma-, or beta- irradiation [22], is described in the literature in detail. These techniques allow for precise and quantitative control over radical formation, making them valuable tools for mechanistic studies. However, their application is limited by accessibility and costs, as they require specialised infrastructure, which is unavailable in most research environments.

We recognised a need within the AEM research community for basic ex situ degradation testing methods that are robust, affordable, and accessible for standard laboratories. These should generate radical species representative of those produced in AEM FCs or ELs.

We adapt two known photochemical methods to generate radical species [23,24]. The effect of these treatments on AEMs is characterised. Both approaches rely on equipment that is either inexpensive or readily available in standard synthetic laboratories, making them highly accessible. Their practical applicability for ex situ degradation studies will be demonstrated and discussed based on three commercially available, state-of-the-art AEMs: PiperION^®^-40 (PiperION), FM-FAA-3-PK-75 (Fumasep), and PNB-R45 (Polynorbornene).

## 2. Experimental Section

### 2.1. Chemicals and Reactants

Benzoic acid (>99.9%, EMSURE^®^ ACS, Reag. Ph Eur), NaOH (>99%, pellets for analysis EMSURE^®^), potassium nitrite (ACS reagent, ≥96.0%), and anatase titanium(IV) dioxide (≥99%, 325 mesh) were purchased from Sigma Aldrich. The AEMs used in this study, PiperION^®^-40 (PiperION), FM-FAA-3-PK-75 (Fumasep), and PNB-R45 (Polynorbornene), were purchased from Fuel Cell store (Bryan, TX 77807, USA). AEMs were exchanged into the hydroxide form following the procedures of the supplier. Samples with 3.5 × 1 cm^2^ size were cut. During the irradiation experiments, 365 nm light sources with a rated power of 3 W (Alonefire SV98-365nm, Shenzen ShiWang Technology Co., Ltd., Guangdong, China) were used, with the charging cable continuously connected. The light source was mounted on top of 25 mL transparent glass vials in such a way that it was positioned 1 cm from the surface of the solution. The irradiance at the irradiation position was determined in-house using a ThorLabs S120C detector (Bergkirchen, 85232 Germany) and was found to be 110 ± 6 mW cm^−2^.

### 2.2. Methods and Equipment

Small molecule study: In the experiments involving potassium nitrite, aqueous solutions containing 4.5 mM BA in 0.1 mM NaOH (pH 10) and varying potassium nitrite concentrations of 0.6–2 mM were irradiated for 30, 60, 90, or 135 min at 365 nm at 80 °C. In the experiments involving TiO_2_, we irradiated at 80 °C aqueous solutions containing 4.5 mM BA, 0.1 mM NaOH (pH 10) and 0.1–1 mg mL^−1^ anatase TiO_2_ at 365 nm for 30, 60, 90, and 135 min.

AEM study: Before the irradiation experiments, commercial AEMs were converted to the hydroxide form by ion-exchanging three times in 1 M KOH and subjected immediately to irradiation to minimise the ion-exchange to carbonate form. The AEMs were placed in a 10 mL solution of 2 mM nitrite, 0.1 mM NaOH, and 0.333 M Na_2_SO_4_, or a 10 mL suspension of 1 mg mL^−1^ TiO_2_ in 0.1 mM NaOH and 0.333 M Na_2_SO_4_, and irradiated at 365 nm for 30, 60, 90, and 135 min. The solutions were stirred at 80 °C at 400 rpm using a 1 cm Teflon-coated stirring bar. The closed, but not sealed, vials were placed in the pre-heated water bath. Following the irradiation, the AEMs were placed in 1 M KOH for 1 h, and then washed successively in water (three times) and used directly to measure ion-exchange capacity.

Ion-exchange capacity (IEC) was measured at room temperature by back titration of the membranes with 0.01 M KOH. The AEM samples were placed into 20 mL 1 M KOH solution for 1 h, washed successively with water (three times), and placed in 10 mL 0.01 M HCl solution for at least 4 h. Finally, the solutions were titrated with a standard 0.01 M KOH solution using a TitroLine^®^ 5000 automatic titrator. The samples were dried in the oven at 80 °C overnight and the dry weight was measured for the membranes in chloride form. The IEC (expressed as mmol g^−1^ dry polymer) was calculated according to Equation (6), where the dry membrane weight (in hydroxide form) was calculated via Equation (7).

(6) $$\mathrm{IEC}=\frac{V_{HCl}*C_{HCl}-V_{KOH}*C_{KOH}}{m_{dry}}$$

(7) $$m_{dry}={m_{dry,Cl-form}-(V}_{HCl}*C_{HCl}-V_{KOH}*C_{KOH})*(M_{Cl-}-M_{HO-})$$

UV-vis measurements were performed using a Thermo Scientific Evolution 220 UV apparatus. An automatic background subtraction was done prior to data analysis. Fourier-Transform Infrared Spectroscopy (FT-IR) measurements were performed using a Bruker vortex 80v apparatus. Measurements were performed under vacuum to suppress water absorption of the AEMs. Every measurement consisted of 20 scans that were averaged. Scanning was conducted from 400 to 4000 cm^−1^ with a spectral resolution of 2 cm^−1^. An automatic background subtraction was carried out prior to data analysis. Changes in the chemical structure of PiperION samples were analysed by ^1^H nuclear magnetic resonance (NMR, AVANCE NEO 400 MHz, Bruker, Ettlingen, 76275 Germany) spectroscopy. The sample was dissolved in 500 µL DMSO-d_6_ and 2 µL trifluoroacetic acid was added. Chemical shifts were referenced to the DMSO-d_6_ solvent signal of 2.50 ppm.

## 3. Results and Discussion

### 3.1. Photochemical Generation of Radicals

The speciation and reactivity of radicals relevant to AEM FCs and ELs depends on pH. The p*K*_a_ of HO^•^ is 11.9 [24]; therefore, both its protonated and deprotonated forms, O^•−^, are present under the alkaline conditions of an AEM FC or EL, with the latter dominating. In contrast, the p*K*_a_ of HOO^•^ is 4.8 [25]; thus, under strongly alkaline conditions, it is present in its deprotonated form O_2_^•−^. We focus, therefore, on the selective production of HO^•^/O^•−^ and O_2_^•−^ in the presence of AEMs. The photolysis of nitrite produces HO^•^ (Reaction (8)).

NO_2_^−^ + H_2_O + *hν* → HO^•^ + NO^•^ + OH^−^ (8)

A critical review of the corresponding photochemistry was published by Mack and Bolton [26]. Takeda et al. recently showed a practical application of that reaction using dilute aqueous solutions of nitrite and a 365 nm UV-LED [23]. The production rate of HO^•^ was estimated by the reaction with terephthalate, a highly sensitive and water-soluble fluorescent probe. Nitrite concentrations were limited to the micromolar range because, while nitrite serves as a photochemical source of hydroxyl radicals (Reaction (8)), it also acts as a highly efficient HO^•^ scavenger (Reaction (9)), with a reported rate constant of *k* = 8 × 10^9^ M^−1^s^−1^ [27].

NO_2_^−^ + HO^•^ → NO_2_^•^ + OH^−^ (9)

Maintaining nitrite concentrations in the micromolar range minimises the effect of Reaction (9). However, it also lowers the maximum achievable steady-state concentration of HO^•^, as photolysis—Reaction (8)—is proportional to the absorption of photons. The latter is proportional to the availability and molar absorptivity of nitrite as a photochemical precursor.

The stress-test of AEMs, on the other hand, requires elevated concentrations of HO^•^. We used 4.5 mM benzoic acid (BA) to probe for photochemically formed hydroxyl radicals (Reaction (10)) and observed the yield of Reaction (10) as a function of the nitrite concentration, which varied between 0.1 mM and 2 mM.

BA + HO^•^ → Products (10)

We calculated the pseudo-first-order rate constants (*k’*) for the reactions of hydroxyl radicals with nitrite and with BA by multiplying the reported second-order rate constants (*k*) with the respective solute concentrations. The resulting *k’* values are proportional to the production rates of the respective processes, and their ratio is proportional to the relative product yields of the competing Reactions (9) and (10) under various conditions (Table 1).

While approximately 92% of the formed HO^•^ radicals react with BA at a low nitrite concentration of 0.1 mM, only 36% will do so at a high nitrite concentration of 2 mM. The higher nitrite concentration significantly increases the production rate of HO^•^ radicals (up to 20 times), which also increases BA degradation despite the lower relative yield of Reaction (8).

**Figure 1:**
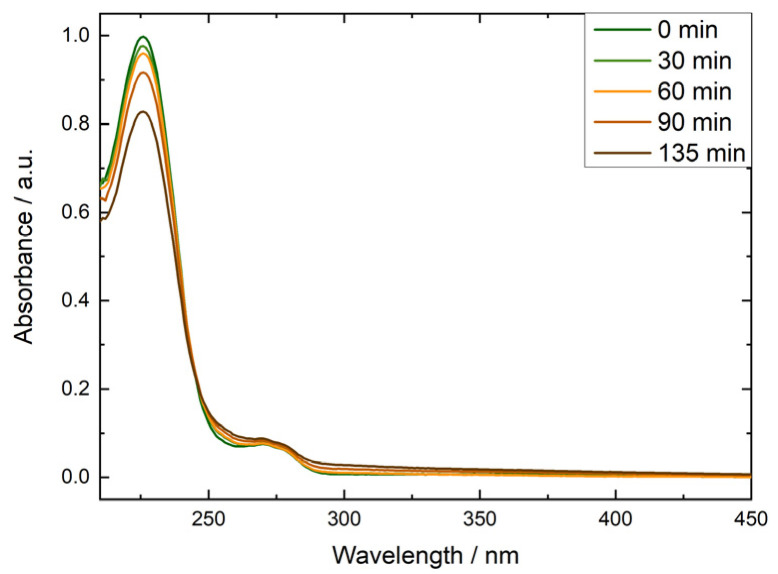
Absorption spectra after 0, 30, 60, 90, and 135 min of irradiation at 80 °C, normalised to the maximum absorbance of BA, measured in 4.5 mM benzoic acid solutions that contained 0.1 mM NaOH and 2 mM potassium nitrite.

**Figure 2:**
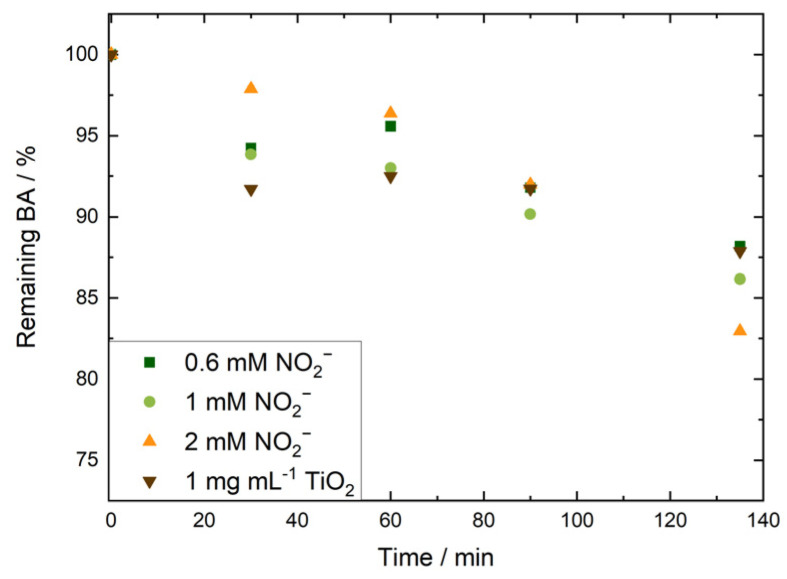
Change in absorbance at 227 nm observed in irradiated 4.5 mM benzoic acid solutions that contained 0.1 mM NaOH and 0.6 (orange triangles), 1 (green circles) or 2 mM (green squares) potassium nitrite or 1 mg mL^−1^ TiO_2_ (brown triangles). Irradiation took place at 80 °C, and UV-vis was recorded at room temperature. Measurements were performed in triplicate (averages shown).

The reaction model was tested using 4.5 mM BA in 0.1 mM NaOH (pH 10) and nitrite concentrations in the range of 0.6–2 mM. Experiments were performed under continuous irradiation at 365 nm. To selectively focus on radical-induced degradation, with limited influence from HO^−^-induced degradation, irradiations were conducted at 80 °C and pH 10. BA degradation was monitored by its absorbance at 227 nm (Figure 1 and Figure 2).

The formation of HO^•^ can be assessed by photochemical probes via the quantification of specific reaction products [28], such as hydroxylated derivatives of BA. However, this method may underestimate HO^•^ formation if multiple parallel reaction pathways are present and the product concentrations are low. A more reliable, albeit less selective, alternative is to monitor the overall consumption of BA.

In all cases, we observed a decrease in BA concentration over time. At shorter irradiation times, BA degradation was larger at lower nitrite concentrations, and after 135 min of continuous irradiation, the average degree of degradation increased with increasing nitrite concentration (Figure 2). To inflict appreciable damage to commercial AEMs, we chose to use a higher nitrite concentration of 2 mM. The controls showed no BA degradation in the absence of irradiation or nitrite.

Not only HO^•^ but also O_2_^•−^ is relevant for the degradation of AEM systems. Since nitrite irradiation forms HO^•^ and NO^•^, we looked for alternative approaches that generate O_2_^•−^ instead. We adapted a photocatalytic method that forms radicals from TiO_2_ suspension [24]. Various ROS are generated in photocatalytic processes on the TiO_2_ surface when exposed to UV irradiation (Reactions (11)−(13)). Conduction band electrons react with dissolved oxygen forming superoxide radicals, and valance band holes react with water to produce HO^•^. Radical generation is dependent on the TiO_2_ crystal phase. Anatase type TiO_2_ is more efficient at radical generation than rutile due to better charge separation and the enhanced absorption of intermediate species [29].

TiO_2_ + *hν* → e^−^_CB_ + h^+^_VB_ (11)

e^−^_CB_ + O_2_ → O_2_^•−^ (12)

h^+^_VB_ + H_2_O → HO^•^ + H^+^ (13)

Irradiation with a 365 nm (3.4 eV) light source is sufficient to form radicals in a suspension of anatase-phase TiO_2_, which has a band gap of approximately 3.2 eV [30]. Accordingly, we irradiated aqueous solutions containing 4.5 mM BA, 0.1 mM NaOH (pH 10), and anatase TiO_2_ at 365 nm for 30, 60, 90, and 135 min at 80 °C. At an optimised TiO_2_ concentration of 1 mg mL^−1^, time-dependent degradation of BA was observed (Figure 2). In subsequent experiments, we implemented the two methods to evaluate the stability of commercial AEMs against radical attack.

### 3.2. Radical-Induced Degradation of AEMs

**Figure 3:**
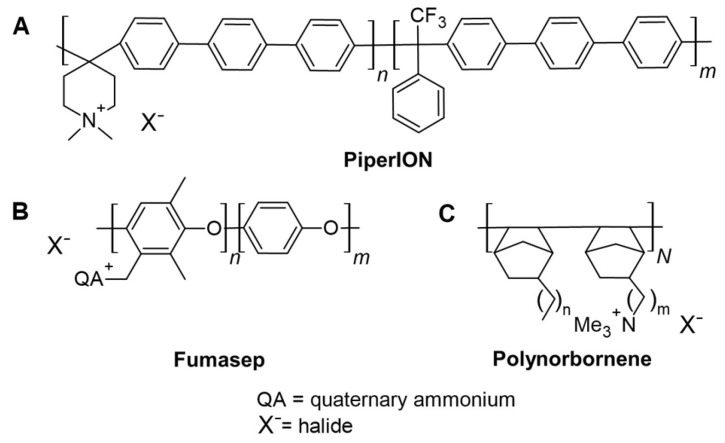
Reported structures of the AEMs investigated in this study, (**A**): PiperION, (**B**): Fumasep, and (**C**): Polynorbornene.

To evaluate and compare the two photochemical methods for studying the radical-induced degradation of AEMs, we selected three commercially available, state-of-the-art AEMs: PiperION, Fumasep, and Polynorbornene (Figure 3). The structural diversity of these membranes formed the basis to demonstrate the broad applicability of the methods. While PiperION and Fumasep feature aromatic backbones [31,32], Polynorbornene is based on a non-aromatic structure [33]. Additionally, Fumasep and Polynorbornene incorporate mechanical reinforcement, polyether ether ketone (PEEK), and polyolefin (PO), respectively. In contrast, PiperION is not reinforced.

### 3.3. Practical Considerations

Before subjecting the AEMs to degradation tests, all samples were converted to the hydroxide form by ion-exchanging three times in 1 M KOH. It is important to note that when membranes are in the halide form, common for commercial AEMs such as Polynorbornene or Fumasep, the generated HO^•^ radicals react diffusion controlled with the halides via Reactions (14) and (15) [34,35]. PiperION is shipped in the bicarbonate form. The reported rate constant for the reaction between carbonate and HO^•^ (Reaction (16)) is one order of magnitude slower [36]. Consequently, a portion of the generated HO^•^ will be consumed in these side reactions, reducing its availability for the primary reaction pathway. Formed X_2_^•−^ and carbonate anion radicals are potent oxidants; however, they are not relevant for FCs if operated in CO_2_-free air, or for ELs if operated using carbonate-free KOH solutions. The authors note that seawater electrolysis and AMEFC tests using regular air are in the centre of increased attention, where X_2_^•−^ and carbonate anion radicals could be of relevance.

Br^−^ + HO^•^ → Br^•^ + HO^−^ *k*
= 1.1 × 10^10^ M^−1^s^−1^ (14)

Br^−^ + Br^•^ → Br_2_^•−^ *k*
= 1.2 × 10^10^ M^−1^s^−1^ (15)

CO_3_^2−^ + HO^•^ → CO_3_^•−^ + HO^−^ *k*
= 4 × 10^8^ M^−1^s^−1^ (16)

Our work focuses on studying the detrimental effects of the most abundant radicals, HO^•^/O^•−^ and O_2_^•−^. Accordingly, we performed all experiments with AEMs in the hydroxide form. Radical reactivity is known to be influenced by ionic strength [37]. Therefore, we used 0.333 M sodium sulphate to establish the same ionic strength as at pH 14, typical of the environment of ex situ stability tests of AEMs [12]. The authors note that the pH of the operating environment for AEM-based devices is typically in the range of pH = 10–14.

Although the reaction rate constants between hydroxide and HO^•^ are also relatively high (Reaction (17)), the reaction product is O^•−^, i.e., the deprotonated form of HO^•^ that dominates under the highly alkaline conditions (pH > 12) [38].

HO^−^ + HO^•^ → H_2_O + O^•−^ *k*
= 1.2 × 10^10^ M^−1^s^−1^ (17)

Next, we reacted the solution-immersed AEMs with primary radicals that were produced by irradiating the solution of 2 mM nitrite, 0.1 mM NaOH, and 0.333 M Na_2_SO_4_, or a suspension of 1 mg mL^−1^ TiO_2_ in 0.1 mM NaOH and 0.333 M Na_2_SO_4_ at 365 nm for 30, 60, 90, and 135 min.

Following irradiation, we performed a visual inspection of the AEM samples to assess their stability against radical attack (Figures S1–S3). We found that the colour of the Fumasep samples darkened after prolonged irradiation with both methods (Figure S1). In contrast, a colour change was observed for both the Polynorbornene and PiperION AEM samples only in the cases of the irradiated nitrite-containing solutions (Figures S2 and S3).

Both the chemical and mechanical degradation of ion-exchange membranes are strongly influenced by the presence of reinforcements and the material thickness. Thicker membranes generally exhibit a lower degree of degradation, as radicals penetrate less deeply, resulting in slower degradation rates. Additionally, reinforced membranes tend to better resist both mechanical and chemical degradation [39].

Accordingly, when we examined changes in the IEC of the AEMs subjected to degradation tests, we observed a gradual decrease in the IEC across all methods and membrane samples (Table 2). However, in the case of Fumasep, degradation was significantly suppressed. This can be attributed to the fact that this AEM was both reinforced and thicker than the other tested membranes, with a dry thickness of 75 µm compared to 40 µm for PiperION and 45 µm for Polynorbornene. We found that Polynorbornene lost approximately 17% and 22% of its IEC after 135 min of continuous irradiation of the nitrite- and TiO_2_-containing solutions, respectively. In contrast, PiperION exhibited IEC losses of approximately 18% and 46% under the same conditions. It was surprising that the degradation of PiperION showed an “induction phase” with the TiO_2_ model. Up to 90 min of irradiation, there was almost no change in the IEC. However, during the next 45 min, there was a loss of almost 50%. This observation was confirmed several times. We note that the reinforcement present in Fumasep and Polynorbornene samples may effectively compete with the ionomer constituents for the generated radicals, which is expected to influence the measured IEC values.

**Figure 4:**
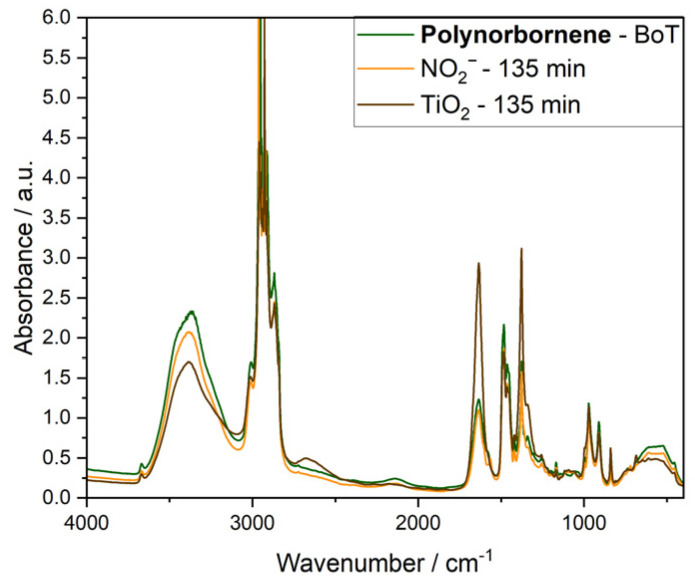
Transmission FT-IR spectra of Polynorbornene AEM samples before (BoT, green line) and after irradiation at 365 nm of the solution of 2 mM nitrite, 0.1 mM NaOH, and 0.333 M Na_2_SO_4_ for 135 min (orange line), and the suspension of 1 mg mL^−1^ TiO_2_ in 0.1 mM NaOH and 0.333 M Na_2_SO_4_ for 135 min (brown line).

To qualitatively assess AEM degradation, we compared the FT-IR spectra recorded before (BoT) and after 135 min of degradation (Figure 4, Figures S4 and S5).

Consistent with the small change in the IEC (Table 2), no significant structural changes could be observed in the reinforced Fumasep samples following the degradation tests (Figure S4). It is important to note that FT-IR measurements are semi-quantitative, and structural changes at the few-percent level may be difficult to detect.

In contrast, the Polynorbornene samples exhibited structural changes, particularly in the absorbance bands associated with C–N stretching vibrations, specifically at 836, 910, 970, and 1076 cm^−1^ [33] (Figure 4). Additionally, changes in the peaks at 1486, 1637, 2146, and 2870 cm^−1^ suggest oxidative degradation of the polymer backbone and side chains, likely involving the formation of unsaturated and carbonyl-containing species. Both the Fumasep and Polynorbornene samples were insoluble in common deuterated solvents, which precluded quantitative NMR analysis.

In the case of PiperION AEMs, increased absorbance at 1633, 1380, 2657, and 2940 cm^−1^ indicates oxidative degradation. The band at 1633 cm^−1^ likely corresponds to the formation of carbonyl-containing species, while the increase at 2940 cm^−1^ may indicate ring-opening of the piperidinium head groups. Notably, significant structural changes were observed at these frequencies in samples irradiated in the presence of TiO_2_ (Figure S5), whereas samples irradiated in the presence of nitrite showed minimal spectral changes.

**Figure 5:**
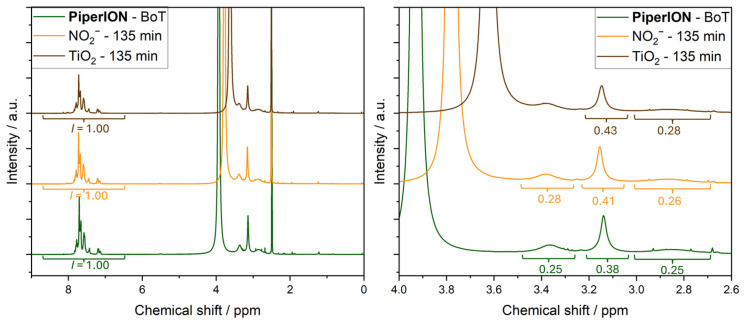
NMR spectra of PiperION AEM samples before (BoT, green line) and after irradiation at 365 nm of the solution of 2 mM nitrite, 0.1 mM NaOH, and 0.333 M Na_2_SO_4_ for 135 min (orange line), and the suspension of 1 mg mL^−1^ TiO_2_ in 0.1 mM NaOH and 0.333 M Na_2_SO_4_ for 135 min (brown line). (**Left**): full spectra, (**right**): enlarged aliphatic region.

Next, we performed an NMR analysis to further analyse the degradation of PiperION. The addition of a small amount of TFA served to separate the *N*,*N*-dimethylpiperidinium signals and downfield-shift the water signal. We compared the ratio of the integrals for the piperidinium ring and quaternary ammonium headgroup, with peaks at 2.88, 3.14, and 3.37 ppm, and of the aromatic protons between 6.5 and 8.5 ppm (Figure 5). The overlap of the water signal with the piperidinium protons at 3.4 ppm prevented the integration of this latter peak in the case of the sample that was irradiated in the presence of TiO_2_. We found that the ratio of aliphatic-to-aromatic protons changed in the case of both PiperION samples that were irradiated for 135 min in the presence of nitrite or TiO_2_. This indicates changes for the aliphatic protons, probably related to the degradation of the quaternary ammonium headgroups, in good agreement with the ion-exchange capacity loss in Table 2. Moreover, we observed decreased solubility and pronounced brittleness in both degraded PiperION samples, which may indicate crosslinking [40].

### 3.4. Applicability of Methods to Compare AEMs

While both methods support the study of the radical-induced degradation of AEMs and minimise the influence from HO^−^-mediated pathways, they exhibit limitations. In the case of the irradiation of nitrite-containing solutions, there is effective competition between nitrite and the AEMs for the generated HO^•^ radicals. As a result, nitrite concentrations must be kept relatively low. However, excessively low nitrite concentrations inherently restrict the steady-state concentration of HO^•^ that can be generated, thereby limiting the extent of AEM degradation. In the authors’ opinion, using 2 mM nitrite offers a good balance between minimising competition and maintaining sufficient radical generation for meaningful degradation. We note that at high oxygen concentrations, highly reactive NO_2_^•^ radicals form via Reaction (18) [41]. However, the high ionic strength established in our experiments limited the availability of oxygen and minimised the interference of Reaction (18).

O_2_ + 2 NO^•^ → 2 NO_2_^•^ *k* = 2.1 × 10^6^ M^−1^s^−1^ (18)

In the case of TiO_2_ suspensions, the rate of radical formation upon irradiation depends on both the crystal structure and the particle size [42]. We observed effective radical generation using anatase-type TiO_2_ with mesh 325. Vigorous stirring of the suspension is essential to maintain the photochemical generation of radicals, as insufficient stirring may cause the TiO_2_ particles to sediment, reducing their exposure to light and thus limiting reaction efficiency. In contrast, for radical generation from nitrite solutions, if the nitrite concentration remains uniform, stirring will not affect the quantum yield of radicals. Both methods of radical production show a larger impact on relatively thin AEMs, as our results show that thicker and reinforced membranes tend to degrade less. This is likely due to the limited penetration depth of radicals, which limits degradation in more robust membrane structures. Table 3 compares the advantages and limitations of available radical-generating methods with the two methods described here.

## 4. Conclusions

We implemented two literature methods to study the radical-induced degradation of AEMs. Both methods rely on the photochemical generation of primary radicals: the UV-irradiation of solutions containing nitrite selectively forms HO^•^/O^•−^, while the irradiation of TiO_2_-containing suspensions generates both HO^•^/O^•−^ and O_2_^•−^. After prolonged irradiation, i.e., at higher turnover of radicals, we observed an enhanced degree of membrane degradation in both methods. By performing the degradation tests at pH 10, we succeeded in deconvoluting base- and radical-induced degradation. Our findings may be exploited for benchmarking novel AEMs against radical stability and aid the design of next-generation AEMs that are stable under alkaline conditions and against radical attack.

## Figures and Tables

**Table 1:** Competing reactions for the hydroxyl radicals at different nitrite concentrations.

Reaction	Reactant	*k* /M^−1^s^−1^	Conc/mM	*k*’ /s^−1^	Yield /%
(7)	NO_2_^−^	8 × 10^9^	0.1	8 × 10^5^	8
(8)	BA	2 × 10^9^	4.5	9 × 10^6^	92
(7)	NO_2_^−^	8 × 10^9^	0.6	4.8 × 10^6^	35
(8)	BA	2 × 10^9^	4.5	9 × 10^6^	65
(7)	NO_2_^−^	8 × 10^9^	1	8 × 10^6^	47
(8)	BA	2 × 10^9^	4.5	9 × 10^6^	53
(7)	NO_2_^−^	8 × 10^9^	2	1.2 × 10^7^	64
(8)	BA	2 × 10^9^	4.5	9 × 10^6^	36

**Table 2:** Remaining ion-exchange capacity of the respective AEMs following the degradation tests.

Remaining IEC ^1^/%						
Time/min	Fumasep		Polynorbornene		PiperION	
	NO_2_^−^	TiO_2_	NO_2_^−^	TiO_2_	NO_2_^−^	TiO_2_
0	100 ± 1	100 ± 3	100 ± 1	100 ± 1	100 ± 1	100 ± 1
30	96 ± 2	96 ± 1	92 ± 6	100 ± 3	93 ± 3	97 ± 1
60	90 ± 1	89 ± 3	83 ± 4	97 ± 8	94 ± 2	99 ± 1
90	92 ± 1	89 ± 3	74 ± 12	84 ± 15	86 ± 2	98 ± 2
135	86 ± 2	91 ± 7	83 ± 1	78 ± 15	82 ± 13	54 ± 2

^1^ IECs were normalised to the beginning-of-test IEC of each respective AEM type. Measurements were performed in triplicate.

**Table 3:** Comparison of available radical-generating methods.

Method	Advantage	Limitation
Fenton’s	Established for PEMs, inexpensive	Ferryl radicals form instead of HO^•^/O^•−^, or O_2_^•−^ at pH > 5
EPR	Selective detection of radicals	Expensive, spin traps may react with radicals that are less relevant for AEMs
Radiolysis	Selective formation of radicals	Expensive, can only be used for dissolved compounds
Thermal H_2_O_2_	Inexpensive and available	Low yield of radicals
Nitrite	Selective formation of HO^•^/O^•−^, inexpensive	Nitrite is a source and scavenger of radicals
TiO_2_	Relevant radicals (HO^•^/O^•−^, or O_2_^•−^) form, inexpensive	Vigorous stirring is required to avoid sedimentation of particles

## Data Availability

Data is available from the corresponding author upon reasonable request.

## Supplementary Materials

The following supporting information can be downloaded at https://www.mdpi.com/article/10.3390/membranes15100305/s1: Figures S1–S3: Images of AEM samples at beginning-of-test (BoT) and after irradiation. Figures S4 and S5: Transmission FT-IR spectra of Fumasep and PiperION AEM samples before (BoT) and after irradiation.
