# Supplementary material for: Photochemical Methods to Study the Radical-Induced Degradation of Anion-Exchange Membranes
Source: Membranes (Basel). 2025 Oct 7;15(10):305. doi: 10.3390/membranes15100305 (PMC12566383; doi:10.3390/membranes15100305)
Supplement: Supplementary file 1 [file membranes-15-00305-s001.zip › membranes-3880953-supplementary.pdf]

Photochemical Methods to Study the Radical Induced Degradation of Anion-Exchange Membranes

Panna Solyom,[a] Thomas Nauser,[b] and Tamas Nemeth*[a]

a Department of Sustainable Energy Technology, SINTEF Industry, 7034 Trondheim, Norway;

b Laboratory of Inorganic Chemistry, ETH Zurich, Vladimir-Prelog-Weg 1, 8093 Zurich, Switzerland

* tamas.nemeth@sintef.no

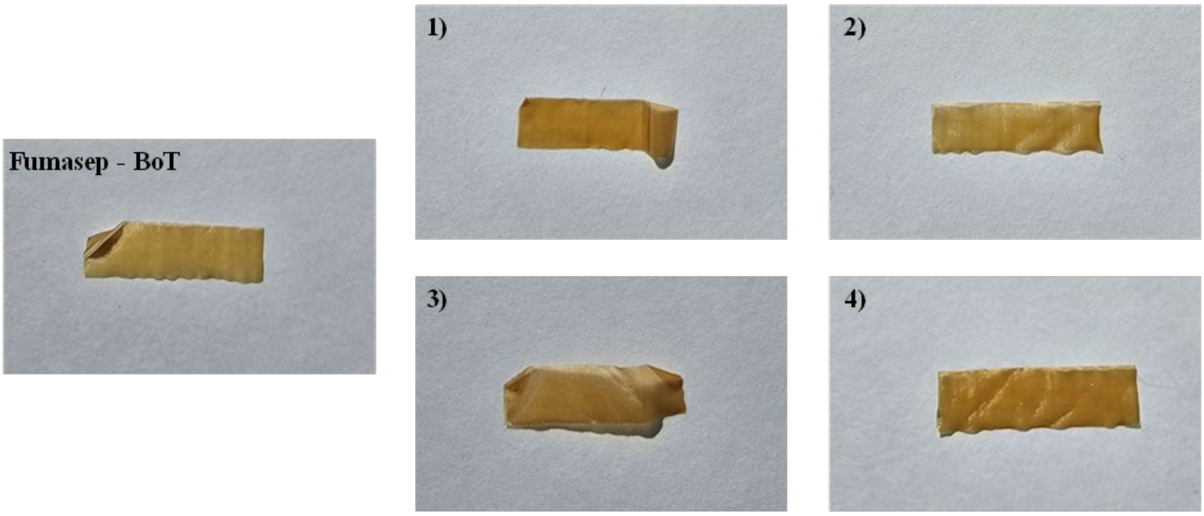

Figure S1. Images of FM-FAA-3-PK-75 (Fumasep) AEM samples at beginning-of-test (BoT) and after irradiation at 365 nm of the solution of 2 mM nitrite, 0.1 mM NaOH and 0.333 M Na_2SO_4 for 60 (1) or 135 min (2), or the suspension of 1 mg mL^{-1} TiO_2 in 0.1 mM NaOH and 0.333 M Na_2SO_4 for 60 (3) or 135 min (4). Sample length in the photos is 3.5 cm.

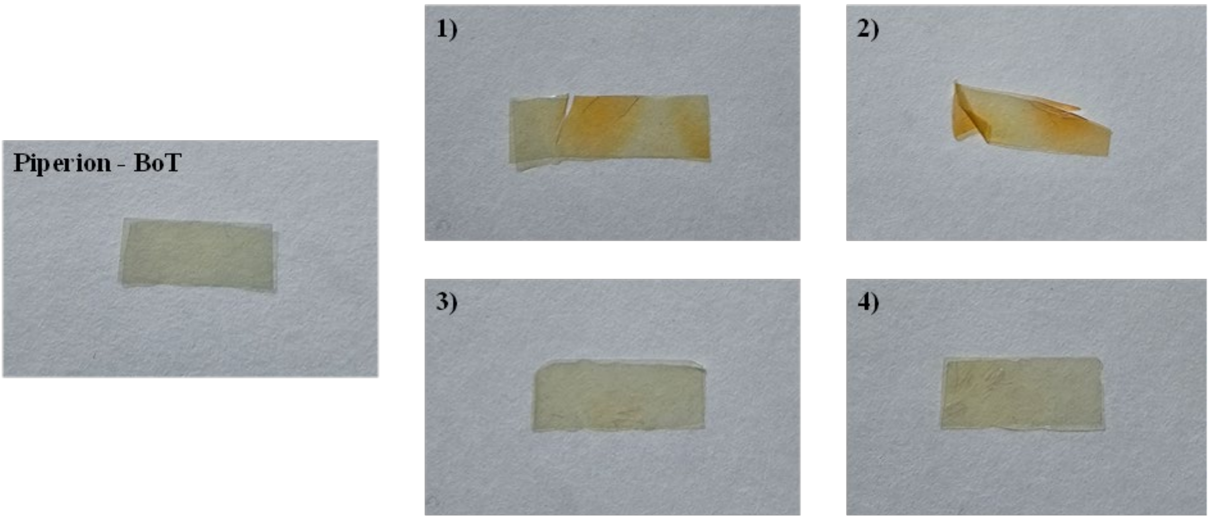

Figure S2. Images of PiperION®-40 (Piperion) AEM samples at beginning-of-test (BoT) and after irradiation at 365 nm of the solution of 2 mM nitrite, 0.1 mM NaOH and 0.333 M Na_2SO_4 for 60 (1) or 135 min (2), or the suspension of 1 mg mL^{-1} TiO_2 in 0.1 mM NaOH and 0.333 M Na_2SO_4 for 60 (3) or 135 min (4). Sample length in the photos is 3.5 cm.

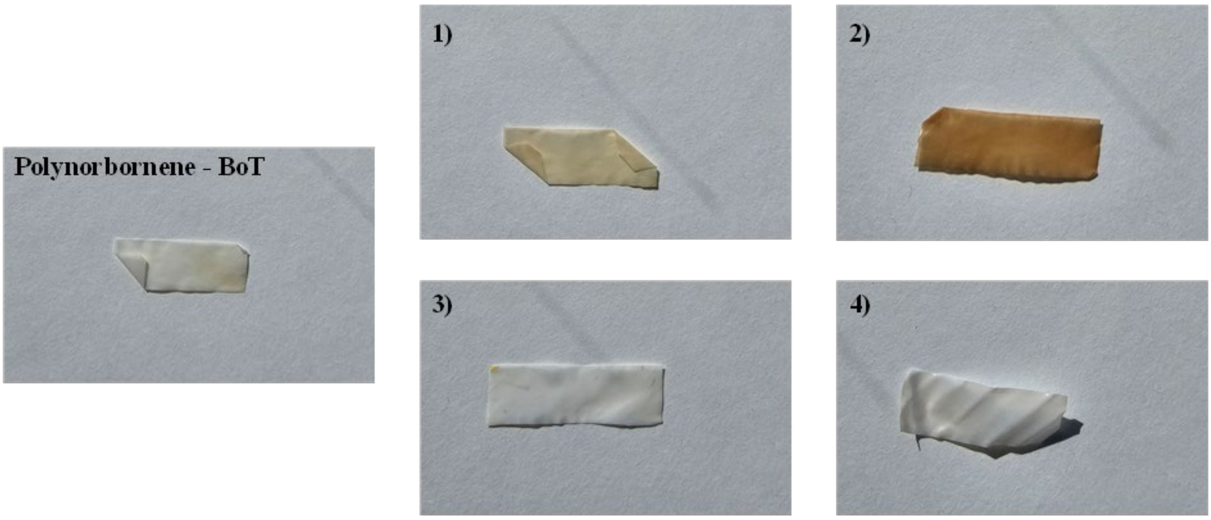

Figure S3. Images of PNB-R45 (Polynorbornene) AEM samples at beginning-of-test (BoT) and after irradiation at 365 nm of the solution of 2 mM nitrite, 0.1 mM NaOH and 0.333 M Na_2SO_4 for 60 (1) or 135 min (2), or the suspension of 1 mg mL^{-1} TiO_2 in 0.1 mM NaOH and 0.333 M Na_2SO_4 for 60 (3) or 135 min (4). Sample length in the photos is 3.5 cm.

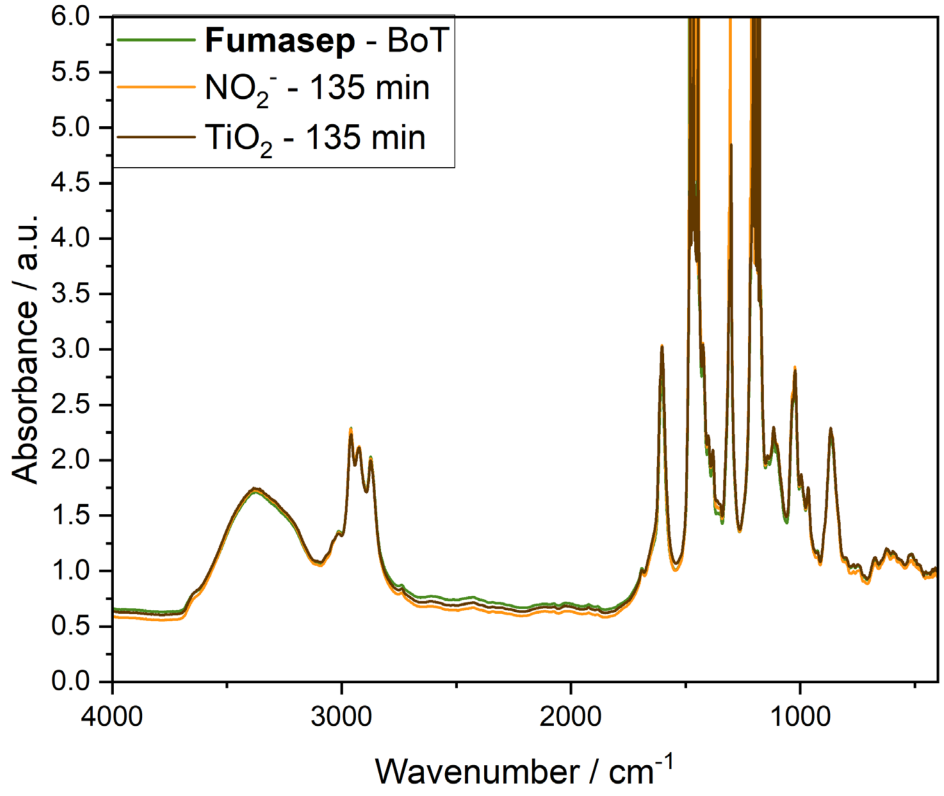

Figure S4. Transmission FT-IR spectra of Fumasep AEM samples before (BoT, orange line) and after irradiation at 365 nm of the solution of 2 mM nitrite, 0.1 mM NaOH and

0.333 M Na_2SO_4 135 min (green line), or the suspension of 1 mg mL^{-1} TiO_2 in 0.1 mM NaOH and 0.333 M Na_2SO_4 for 135 min (brown line)

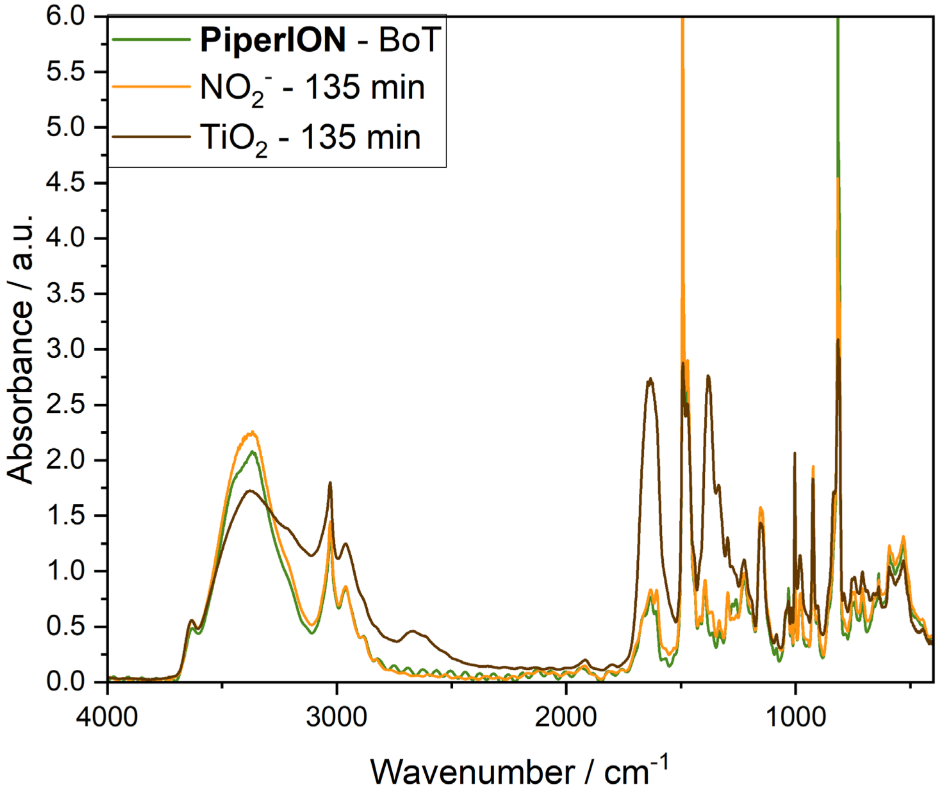

Figure S5. Transmission FT-IR spectra of PiperION AEM samples before (BoT, orange line) and after irradiation at 365 nm of the solution of 2 mM nitrite, 0.1 mM NaOH and 0.333 M Na_2SO_4 135 min (green line), or the suspension of 1 mg mL^{-1} TiO_2 in 0.1 mM NaOH and 0.333 M Na_2SO_4 for 135 min (brown line)

Details on calculating the ionic strength

In the degradation tests of AEMs, we have used solutions containing 2 mM KNO_2, 0.1 mM NaOH and 0.333 M Na_2SO_4 or a suspension that contained 1 mg mL^{-1} TiO_2, 0.1 mM NaOH and 0.333 M Na_2SO_4.

Ionic strength (I) was calculated using the formula: $I = \frac{1}{2} \Sigma c_i \cdot z_i^2$

Where: c_i is the concentration of ion i (M) and z_i is the charge of ion i

1. Potassium Nitrite (KNO_2) dissociates as:

$KNO_2 \rightarrow K^+ + NO_2^-$

Each at 2 mM = 0.002 M

2. Sodium Hydroxide (NaOH) dissociates as:

$NaOH \rightarrow Na^+ + OH^-$

Each at 0.1 mM = 0.0001 M

3. Sodium Sulfate (Na_2SO_4) dissociates as:

$Na_2SO_4 \rightarrow 2\ Na^+ + SO_4^{2-}$

$Na^+ = 2 \times 0.333$ M = 0.666 M

$SO_4^{2-} = 0.333$ M

Using the formula:

$I = 0.5 \times [\ (0.002 \times 1^2) + (0.002 \times 1^2) + (0.0001 \times 1^2) + (0.0001 \times 1^2) + (0.666 \times 1^2) + (0.333 \times 2^2)\ ] = 1.0511$ M

Therefore, the ionic strength of the solution is approximately 1.05 M.

In case of the TiO_2-containing suspensions, only 0.1 mM NaOH + 0.333 M Na_2SO_4 needs to be considered:

Dissociation:

$NaOH \rightarrow Na^+ + OH^- \rightarrow$ 0.0001 M of each ion

$Na_2SO_4 \rightarrow 2\ Na^+ + SO_4^{2-} \rightarrow$ 0.333 M gives: 0.666 M Na^+ and 0.333 M SO_4^{2-}

Total ion concentrations:

- Na^+: 0.0001 + 0.666 = 0.6661 M

- OH^-: 0.0001 M

- SO_4^{2-}: 0.333 M

Ionic strength calculation:

$I = \frac{1}{2}\ (0.6661 \cdot 1^2 + 0.0001 \cdot 1^2 + 0.333 \cdot 2^2) = 0.9991$ M

A typical electrolyte concentration of AEM water electrolysers is 1 M KOH:

Dissociation:

$KOH \rightarrow K^+ + OH^- \rightarrow$ 1 M of each ion, therefore, $I = \frac{1}{2}\ (1 \cdot 1^2 + 1 \cdot 1^2) = \frac{1}{2}\ (2) = 1.0$ M
